# Comparison of Quality Measures From US Hospitals With Physician vs Nonphysician Chief Executive Officers

**DOI:** 10.1001/jamanetworkopen.2022.36621

**Published:** 2022-10-13

**Authors:** Helen See, Lacey Shreve, Sarah Hartzell, Sarah Daniel, Anthony D. Slonim

**Affiliations:** 1 Renown Health, Reno, Nevada; 2 School of Public Health, University of Nevada, Reno; 3 Mississippi Delta Family Medicine, Greenville; 4 Medicine and Pediatrics, University of Nevada, Reno

## Abstract

**Importance:**

Patient experience and patient safety are 2 major domains of health care quality; however empirical data on the association of physician vs nonphysician chief executive officers (CEOs) with public and private quality measures are rare but critical to evaluate as hospitals increasingly seek out physician CEOs.

**Objectives:**

To evaluate whether there is an association of CEO background with hospital quality and to investigate differences in hospital characteristics between hospitals with a physician CEO vs those with a nonphysician CEO.

**Design, Setting, and Participants:**

This cross-sectional study used 2019 data from 3 sources (ie, the American Hospital Association [AHA] Annual Survey, the Hospital Consumer Assessment of Healthcare Providers and Systems [HCAHPS], and the Leapfrog Hospital Safety Grades) to identify statistical differences in hospital characteristics and outcomes. Data were analyzed from April to December 2021 .

**Main Outcomes and Measures:**

Multivariable ordinal logistic regression was used to examine the association of physician CEOs with hospital quality assessment outcomes while controlling for other confounding factors. Characteristics from the AHA Annual Survey database were assessed as potential confounders, including hospital control, bed size, region, teaching status, and patient volume.

**Results:**

The AHA database contained 6162 hospitals; 1759 (29%) had HCAHPS ratings, 1824 (30%) had Leapfrog grades, and 383 (6%) had physician CEOs. A positive Spearman correlation coefficient was found between physician CEOs and HCAHPS patient willingness to recommend the hospital (ρ = 0.0756; *P* = .002), but the association between CEO medical background and Leapfrog safety grades or HCAHPS ratings did not reach a level of significance in the multivariable ordinal logistic regression models.

**Conclusions and Relevance:**

In this study, a positive correlation was found between physician CEOs and HCAHPS patient willingness to recommend the hospital, but the multivariable analysis did not find an association between hospital physician CEOs and the examined quality and safety outcomes.

## Key Points

**Question:**

Is having a physician serve as chief executive officer (CEO) associated with hospital quality assessment, indicated by Hospital Consumer Assessment of Healthcare Providers and Systems (HCAHPS) ratings and Leapfrog Hospital Safety Grades?

**Findings:**

In this cross-sectional study of 6162 US hospitals, approximately 30% of hospitals participated in 2019 HCAHPS and Leapfrog hospital surveys. A positive association was found between physician CEOs and HCAHPS patient willingness to recommend the hospital, but the multivariable model found no significant association between hospitals led by a physician CEO and higher HCAHPS ratings or Leapfrog grades.

**Meaning:**

This study found a correlation between physician leadership and a patient’s willingness to recommend the hospital; however, no significant association between CEO background and any quality measures were identified in the multivariable models.

## Teaser

This cross-sectional study evaluates whether there is an association of hospital chief executive officer (CEO) background with hospital quality and investigates differences in hospital characteristics between hospitals with a physician CEO vs those with a nonphysician CEO.

## Introduction

While efforts to improve health care quality date back to the 1980s, the Institute of Medicine’s (IOM) landmark reports focused on and prioritized the study of health care quality improvement in the United States.^1,2,3,4^ Today, hospitals dedicate significant efforts to improve both patient experience and patient safety, 2 of the major pillars highlighted by the IOM. In the United States, the Centers for Medicare & Medicaid Services (CMS) developed the Hospital Consumer Assessment of Healthcare Providers and Systems (HCAHPS) survey in 2002 to measure patients’ perceptions of their hospital experience.^5^ Additionally, the Leapfrog Group, a national nonprofit organization, developed the Leapfrog Hospital Safety Grade, which has become a nationally accepted benchmark for patient safety.

Administrative physicians have a unique opportunity to apply their subject-matter expertise to complex challenges that can benefit both the patient and the overall health system. Chief executive officers (CEOs) with a background in clinical care are increasingly sought by hospitals to operationalize strategic direction in quality performance of value-based care, such as patient safety and satisfaction.^6,7,8,9,10^ Few empirical studies or meta-analyses have investigated the association between CEO educational background and quality outcome measures.^9,11,12,13,14,15,16,17,18^ Consequently, the primary research question of this study is to assess whether physician background was associated with quality assessment outcomes, represented by HCAHPS ratings and Leapfrog safety grades.

## Methods

Per the Common rule, this study did not need institutional review board approval or informed consent because it was nonhuman participant research. All data used are publicly available. The reporting followed the Strengthening the Reporting of Observational Studies in Epidemiology (STROBE) reporting guideline.

Data are a linkage of 3 sources, public and proprietary, combined with data collected by the authors. The source population was derived from the 2019 American Hospital Association (AHA) Annual Survey database, and quality assessment outcomes were linked to publicly available data, including the CMS 2019 HCAHPS survey and the Leapfrog Group’s 2019 Hospital Safety Grades. Not all hospitals in the AHA database had outcome measures in both HCAHPS and Leapfrog. Because physician CEOs are rare and to maximize the exposure, we chose to analyze 2 separate samples of AHA hospitals, one consisting of all hospitals with HCAHPS outcomes and one consisting of all hospitals with Leapfrog outcomes. Hospitals in the AHA database were matched by hospital name, state, and county to their respective quality metrics from each reporting entity. The authors collected the 2019 spring and fall Leapfrog grades from their website.^19^ We excluded hospitals with a bed size of 6 to 24, hospitals located in territories outside the 50 US states, hospitals operated under federal government control, and children’s and specialty hospitals.

### Data Sources and Participants

The AHA Annual Survey Database is a proprietary hospital database for peer comparisons of health services research produced primarily from the AHA Annual Survey of Hospitals. Participation in the AHA Annual Survey is voluntary and open to all hospitals in the United States and does not require its participants to be a member of the AHA.^20^

The HCAHPS survey is a national, standardized, publicly reported survey of patients’ perspectives regarding their hospital care and experience administered to a random sample of adult discharged patients not restricted to CMS beneficiaries.^5^ All short-term, acute care, nonspecialty hospitals in compliance with the quality assurance guidelines are invited to participate in the HCAHPS data collection, but only hospitals receiving payments from Medicare Part A inpatient prospective payment system (IPPS) are required to participate.^21,22^ More than three-quarters of the nation’s inpatient acute-care hospitals are paid under Medicare’s IPPS.^23^ This analysis uses 2 HCAHPS measures, including the patients’ willingness to recommend rating and the overall summary star rating.

Criteria used to produce the Leapfrog safety grade were selected by a panel of 9 experts using a publicly available scoring algorithm, which combines data from CMS and the Leapfrog Hospital Survey.^24^ All hospitals are encouraged to voluntarily report additional data through the Leapfrog Hospital Survey, but they are not required to participate in the survey to receive a safety grade.^24^ More than half of all acute-care hospitals in the United States are assigned a Leapfrog safety grade but, due to limitations in publicly available data, Leapfrog does not calculate grades for specific hospital types.^25^

### Variables

#### Dependent Variables

There were 2 dependent variables analyzed for both HCAHPS and Leapfrog samples. We chose to analyze the HCAHPS recommended rating because it best reflected the patients’ choice in returning to the specified hospital and/or recommending it to a peer and the summary star rating because it summarizes all 11 HCAHPS ratings into a global rating. HCAHPS ratings are provided to each hospital in an ordinal score from 1 to 5, with 5 being the highest and most desirable rating. The Leapfrog Group provides 2 safety grades per hospital per year (spring and fall), consisting of five ordinal categories ranging from the lowest grade of an F to the highest and most desirable grade of an A.

#### Independent Variables

The primary exposure of interest was the medical background of the individual listed as the hospital CEO in the AHA database in 2019. The CEO’s medical education was identified using a search in LinkedIn and/or Google and dichotomously ascribed as physician if that individual held a medical doctor (MD) or doctor of osteopathy degree or as nonphysician if that individual did not.

Continuous and categorical independent variables from the AHA database were assessed for potential confounding. Continuous variables included total facility admissions, adjusted admissions, total facility inpatient days, adjusted patient days, total facility Medicare discharges, total facility Medicaid discharges, total births (excluding fetal deaths), total surgical operations, emergency department visits, total facility personnel full time equivalent, and adjusted average daily census. Categorical independent variables included state, bed size, a variable to indicate the controlling body over the hospital, and a dichotomous variable to indicate the hospital’s membership in the Council of Teaching Hospitals (COTH).

### Statistical Analysis

Using SAS version 9.4 (SAS Institute Inc) software, we examined the frequency distribution of CEO background, controlling body, bed size, region, and COTH status in the full AHA sample, the HCAHPS sample, and the Leapfrog sample. We used χ^2^ tests to compare the sample distributions with the source population distributions. All significance testing was assessed for an α < .05. Continuous variables were tested for normality, and *P* values from the Kruskal-Wallis test were compared for differences in patient volume across the 5 categories of HCAHPS ratings and Leapfrog grades and included in the eTable in the Supplement. We assessed the correlation between HCAHPS and Leapfrog ordinal outcomes with physician status using a Spearman rank correlation coefficient and *P* values. We assessed the association between each covariate and outcome using bivariate ordinal logistic regression analysis with each independent variable modeling toward the most desirable outcome (rating of 5 or grade of A). Covariates significantly associated with the dependent variables were included in the multivariable logistic regression models and presented in the results.

## Results

Table 1 displays the 2019 AHA source population, comprised of 6162 hospitals, of which 1759 (29%) received an HCAHPS rating and 1824 (30%) received a Leapfrog grade. We found 383 hospitals (6%) led by physician CEOs, of which 131 (7%) had HCAHPS ratings and 166 (9%) had Leapfrog grades. Table 1 shows no significant differences between the distribution of physician status, region, and COTH status in the HCAHPS sample and the source population. In the Leapfrog sample, there was no significant difference from the source population in COTH status distribution.

**Table 1.** Hospital Characteristics by Chief Executive Officer Medical Background

Characteristic	Hospitals, No. (%)								
	AHA survey			HCAHPS sample			Leapfrog sample		
	Physician (n = 383)	Nonphysician (n = 5779)	Total (N = 6162)	Physician (n = 131)^a^	Nonphysician (n = 1628)^a^	Total (n = 1759)	Physician (n = 166)	Nonphysician (n = 1658)	Total (n = 1824)
Controlling body									
Government, nonfederal	64 (16)	1160 (20)	1224 (20)	21 (16)	262 (16)	283 (16)	22 (13)	179 (11)	201 (11)
Nongovernment, not-for-profit	250 (65)	2872 (50)	3122 (51)	101 (77)	979 (60)	1080 (61)	137 (83)	1134 (68)	1271 (70)
Investor-owned, for profit	40 (10)	1565 (27)	1605 (26)	9 (7)	387 (24)	396 (23)	7 (4)	345 (21)	352 (19)
Government, federal	29 (8)	182 (3)	211 (3)	0	0	0	0	0	0
Bed size									
6-24	31 (8)	814 (14)	845 (14)	0	0	0	0	0	0
25-199	176 (46)	3629 (63)	3805 (62)	58 (44)	1070 (66)	1128 (64)	62 (37)	852 (51)	914 (50)
200-399	92 (24)	910 (16)	1002 (16)	42 (32)	372 (23)	414 (24)	54 (33)	532 (32)	586 (32)
≥400	84 (22)	426 (7)	510 (8)	31 (24)	186 (11)	217 (12)	50 (30)	274 (16)	324 (18)
Region									
West	64 (17)	1119 (19)	1183 (19)	25 (19)^a^	377 (23)	402 (23)	25 (15)	335 (20)	360 (20)
Midwest	120 (31)	1560 (27)	1680 (27)	35 (27)^a^	370 (23)	405 (23)	51 (31)	375 (23)	426 (23)
South	102 (27)	2335 (40)	2437 (40)	34 (26)^a^	667 (41)	701 (40)	45 (27)	711 (43)	756 (41)
Northeast	88 (23)	702 (12)	790 (13)	37 (28)^a^	214 (13)	251 (14)	45 (27)	237 (14)	282 (15)
Other territory	9 (2)	63 (2)	72 (1)	0^a^	0	0	0	0	0
COTH Status									
COTH	79 (21)	216 (21)	295 (5)	31 (24)^a^	81 (5)	112 (6)	44 (27)^a^	122 (7)	166 (9)
Non-COTH	304 (79)	5563 (79)	5867 (95)	100 (76)^a^	1547 (95)	1647 (94)	122 (73)^a^	1536 (93)	1658 (91)

Abbreviations: AHA, American Hospital Association; COTH, Council of Teaching Hospitals; HCAHPS, Hospital Consumer Assessment of Healthcare Providers and Systems.

^a^ No significant difference between the sample distribution and the distribution of the source population.

Table 2 displays the HCAHPS and Leapfrog outcomes by physician and nonphysician status along with the Spearman correlation coefficients and *P* values. Overall, 605 hospitals (34%) had HCAHPS recommended ratings of 4 or 5; 601 (34%) had HCAHPS summary star ratings of 4 or 5, 1087 (60%) had Leapfrog grades of A or B in spring 2019, and 1096 (60%) had Leapfrog A grades in fall 2019. HCAHPS recommended rating was the only quality measure to show a significant correlation with hospitals led by physician CEOs (ρ = 0.0756; *P* = .002). Overall, 13 hospitals led by physicians (10%) received a HCAHPS recommended rating of 5, while 80 hospitals (5%) led by nonphysicians received a rating of 5 (Table 2).

**Table 2.** HCAHPS Scores and Leapfrog Grades by Physician Status

Score	HCAHPS Recommended rating, No. (%)		Spearman ρ	*P* value	HCAHPS Summary star rating, No. (%)		Spearman ρ	*P* value
	Physician	Nonphysician			Physician	Nonphysician		
Hospitals, No.	131	1628	NA	NA	131	1628	NA	NA
5	13 (10)	80 (5)	0.0756	.002	7 (5)	83 (5)	–0.005	.84
4	46 (35)	466 (29)			33 (25)	478 (29)		
3	45 (34)	589 (36)			65 (50)	710 (44)		
2	24 (18)	413 (25)			25 (19)	309 (19)		
1	3 (2)	80 (5)			1 (1)	48 (3)		
**Grade**	**Leapfrog spring, No. (%)**		**Spearman ρ**	***P* value**	**Leapfrog fall, No. (%)**		**Spearman ρ**	***P* value**
Hospitals, No.	166	1658	NA	NA	166	1658	NA	NA
A	56 (34)	554 (33)	0.001	.98	66 (40)	572 (35)	0.026	.27
B	43 (26)	434 (26)			41 (25)	417 (25)		
C	56 (34)	565 (34)			43 (26)	554 (33)		
D	11 (7)	98 (6)			16 (10)	109 (7)		
F	0	7 (<1)			0	6 (<1)		

Abbreviations: HCAHPS, Hospital Consumer Assessment of Healthcare Providers and Systems; NA, not applicable.

Preliminary bivariate analyses were conducted, and only the primary exposure of interest and covariates demonstrating a significant association with the outcome were included in the multivariable logistic regression models to obtain the adjusted odds ratios, which are displayed in Table 3. All bivariate results were nonsignificant, except the HCAHPS recommended rating (odds ratio, 1.70; 95% CI, 1.23-2.35; *P* = .001); however, these results were nullified in the multivariable model. After adjusting for covariates, we found no results with a significance level of α < .05 (Table 3).

**Table 3.** Multivariable Ordinal Logistic Regression: AORs by Performance Measure^a^

Hospital characteristics	HCAHPS recommended rating^b^		HCAHPS summary star rating^b^		Leapfrog spring^c^		Leapfrog fall^c^	
	AOR (95% CI)	*P* value	AOR (95% CI)	*P* value	AOR (95% CI)	*P* value	AOR (95% CI)	*P* value
CEO background								
Physician	1.38 (0.98-1.93)	.06	0.97 (0.69-1.37)	.85	1.03 (0.77-1.39)	0.83	1.23 (0.91-1.64)	.18
Nonphysician	1 [Reference]	NA	1 [Reference]	NA	1 [Reference]	NA	1 [Reference]	NA
Controlling body								
Government, nonfederal	2.82 (2.12-3.76)	<.001	3.84 (2.85-5.17)	<.001	0.42 (0.30-0.57)	<.001	0.36 (0.26-0.50)	<.001
Nongovernment, not-for-profit	2.86 (2.28-3.60)	<.001	2.96 (2.34-3.74)	<.001	0.89 (0.72-1.11)	.30	0.90 (0.72-1.12)	.33
Investor-owned, for profit	1 [Reference]	NA	1 [Reference]	NA	1 [Reference]	NA	1 [Reference]	NA
Bed size								
25-199	1 [Reference]	NA	1 [Reference]	NA	NA	NA	NA	NA
200-399	0.76 (0.61-0.93)	.009	0.29 (0.23-0.36)	<.001	NA	NA	NA	NA
≥400	1.12 (0.82-1.53)	.49	0.27 (0.20-0.37)	<.001	NA	NA	NA	NA
Region								
West	1.17 (0.93-1.46)	.186	0.73 (0.58-0.92)	.007	NA	NA	NA	NA
Midwest	1.70 (1.34-2.14)	<.001	2.39 (1.88-3.05)	<.001	NA	NA	NA	NA
South	1 [Reference]	NA	1 [Reference]	NA	NA	NA	NA	NA
Northeast	0.76 (0.58-1.01)	.06	0.86 (0.64-1.14)	.28	NA	NA	NA	NA
COTH Status								
COTH	1.72 (1.13-2.62)	.01	1.29 (0.84-1.99)	.25	NA	NA	NA	NA
Non-COTH	1 [Reference]	NA	1 [Reference]	NA	NA	NA	NA	NA

Abbreviations: AOR, adjusted odds ratio; CEO, chief executive officer; COTH, Council of Teaching Hospitals; HCAHPS, Hospital Consumer Assessment of Healthcare Providers and Systems; NA, not applicable.

^a^ *P* values derived from Wald χ^2^ tests, with a significance level of α < .05.

^b^ Ordinal logistic regression modeling of the association between physician status and a higher HCAHPS rating, controlling for bed size, region, and control.

^c^ Ordinal logistic regression modeling of the association between physician status and a higher Leapfrog grade, controlling for bed size, region, and control.

## Discussion

Results of this study provide exploratory strategies for measuring available data sources for investigating the association between physician leadership at the CEO level and quality outcomes indicated by HCAHPS ratings and Leapfrog grades. We found a correlation between HCAHPS recommended rating and physician CEO status; however, these results were nullified in the multivariable regression analysis for all outcome categories.

In our effort to better understand the association of CEO background with HCAHPS recommended rating, we were unable to find results with statistical significance. Other researchers have found mixed results in evaluating whether physician-led hospitals perform better than nonphysician-led hospitals. Tasi and colleagues^16^ found that physician-led hospital systems had higher quality ratings compared with nonphysician-led hospital systems. Yet, a recent publication by Moores and colleagues^26^ found a significantly higher 30-day cardiovascular mortality rate in physician-led hospitals (80 deaths) when compared with nonphysician led hospitals (72 deaths), but the association was nullified in the multivariate linear regression analysis. The authors suggested that this trend may influence clinical outcomes, yet temporality could not be established because of the study design.^26^ Additionally, the authors noted that future research should include CEOs with 3 years of tenure, which was unaccounted for in our cross-sectional design.^26^

### Limitations

Our study has a number of limitations to consider. First, cross-sectional studies cannot assess causality, and our study considered a limited number of potential confounders. Second, our cross-sectional study design cannot establish temporality, and it has been argued that better quality hospitals have a wider pool of CEO candidates to choose from and may be more likely to seek out MDs as leaders.^27^ Third, our study results are only representative of hospitals that participated in HCAHPS and Leapfrog and cannot be generalized to all US hospitals. We found that HCAHPS ratings and Leapfrog grades were only reported for approximately 30% of hospitals in the 2019 AHA database, which limits the generalizability of our results. We also found that roughly 60% of hospitals graded by Leapfrog in 2019 received a grade of an A or B compared with approximately 33% of hospitals participating with HCAHPS receiving a score of a 4 or 5. Fourth, our study was missing some potentially important confounders, including CEO tenure, and risk adjustment.^28^ Additionally, there may be misclassification of the exposure because exposure data were collected from publicly available internet sources that may or may not be up to date; however, we feel this potential misclassification would be approximately equal across outcome categories, potentially biasing results toward the null.

## Conclusions

The findings of our study failed to identify a statistically significant association between a CEO’s medical background and hospital quality measures, indicated by HCAHPS ratings and Leapfrog grades. Our study described the context of HCAHPS ratings and Leapfrog grades for hospitals in the 2019 AHA database and attempted to aggregate CEO medical background based on publicly available data sources. As health care in the United States becomes increasingly focused on value-based care, complex challenges require solutions at a system level to ultimately improve patient care. Further study is encouraged to better understand the association of the CEO’s medical background with patient satisfaction and safety quality outcomes for US hospitals.

## Notes

Supplement. **eTable.** Descriptive Statistics and Patient Volume Indicators by Hospital Quality Assessment
