# Supplementary material for: Comparison of Quality Measures From US Hospitals With Physician vs Nonphysician Chief Executive Officers
Source: JAMA Netw Open. 2022 Oct 13;5(10):e2236621. doi: 10.1001/jamanetworkopen.2022.36621 (PMC9561952; doi:10.1001/jamanetworkopen.2022.36621)
Supplement: Supplement. — eTable. Descriptive Statistics and Patient Volume Indicators by Hospital Quality Assessment [file jamanetwopen-e2236621-s001.pdf]

Supplemental Online Content

See H, Shreve L, Hartzell S, Daniel S, Slonim AD. Comparison of quality measures from US hospitals with physician vs nonphysician chief executive officers. *JAMA Netw Open.* 2022;5(10):e2236621. doi:10.1001/jamanetworkopen.2022.36621

eTable. Descriptive Statistics and Patient Volume Indicators by Hospital Quality Assessment

This supplemental material has been provided by the authors to give readers additional information about their work.

© 2022 See H et al. *JAMA Network Open.*

eTable. Descriptive Statistics and Patient Volume Indicators by Hospital Quality Assessment Measure

Patient Volume Indicators (Q1-Q3)[1]	HCAHPS Sample (N=1,759)											
	Recommended Rating						Summary Star Rating					
	1	2	3	4	5	p-value	1	2	3	4	5	p-value
**Total Sample[2]**	**N=83**	**N=437**	**N=634**	**N=512**	**N=93**	<.001	**N=49**	**N=334**	**N=775**	**N=511**	**N=90**	<.001
Total facility admissions	6,006 (2,737-9,236)	6,157 (2,819-11,237)	**6,321** (2,542-13,472)	5,945 (1,667-15,045)	1,673 (861-8,186)	<.001	8,637 (6,290-13,415)	**10,169** (5,695-16,142)	7,217 (3,371-14,011)	2,581 (1,224-7,798)	1,117 (724-1,641)	<.001
Adjusted admissions	13,419 (7,355-18,542)	14,618 (8,487-22,678)	**15,580** (8,167-28,199)	15,484 (6,685-32,126)	5,943 (3,726-21,667)	<.001	15,512 (10,657-24,396)	**19,142** (12,947-30,244)	17,128 (9,604-29,592)	9,259 (5,274-20,675)	4,671 (3,473-6,424)	<.001
Total facility inpatient days	28,992 (14,542-52,894)	29,904 (13,688-59,495)	**32,085** (11,790-65,154)	27,642 (7,615-69,919)	6,466 (3,423-35,720)	<.001	**48,249** (32,679-76,005)	45,284 (25,512-78,982)	35,418 (15,718-69,542)	12,610 (4,824-36,283)	4,371 (2,831-8,874)	<.001
Adjusted patient days	66,264 (32,349-97,350)	69,504 (39,366-113,776)	**76,513** (35,127-140,604)	71,573 (29,262-155,204)	25,288 (14,104-98,810)	<.001	87,503 (65,566-113,776)	**94,790** (59,374-145,434)	83,165 (42,722-152,885)	39,570 (21,853-93,085)	19,563 (14,097-46,105)	<.001
Total facility Medicare discharges	2,735 (1,445-4,303)	**3,145** (1,465-5,370)	3,108 (1,315-6,501)	2,940 (829-6,780)	835 (487-4,185)	<.001	3,957 (2,465-5,801)	**4,476** (2,721-7,299)	3,726 (1,683-6,687)	1,231 (646-3,974)	595 (437-839)	<.001
Total facility Medicaid discharges	1,262 (491-2,365)	**1,173** (568-2,514)	1,135 (438-2,760)	904 (286-2,460)	190 (84-659)	<.001	1,807 (1,114-3,886)	**2,037** (1,031-3,926)	1,266 (586-2,888)	455 (188-1,236)	120 (72-253)	<.001
Total births	429 (0-919)	543 (236-1,088)	571 (225-1,364)	**583** (149-1,899)	133 (0-605)	<.001	761 (0-1,162)	**948** (363-1,842)	694 (306-1,459)	320 (98-833)	67 (0-203)	<.001
Total surgical operations	4,048 (2,291-6,323)	4,446 (3,022-7,257)	4,923 (2,739-9,600)	**5,621** (2,389-11,480)	2,598 (1,637-8,890)	0.001	4,571 (3,234-7,680)	**5,911** (3,727-9,648)	5,503 (3,294-10,721)	3,357 (1,818-7,684)	1,987 (1,115-2,712)	<.001
Emergency department visits	25,056 (15,014-44,929)	31,565 (18,778-51,968)	**32,467** (16,785-55,975)	29,771 (13,675-52,273)	10,279 (4,561-35,522)	<.001	33,122 (16,554-50,259)	**43,679** (26,158-65,161)	35,222 (20,845-57,182)	19,627 (10,407-37,182)	7,872 (4,745-12,236)	<.001
Total facility personnel FTE[3]	673 (303-944)	802 (486-1378)	**944** (421-1857)	905 (385-2186)	410 (275-1906)	0.011	862 (700-1548)	**1,179** (651-1978)	1,050 (525-2074)	562 (291-1,293)	321 (231-411)	<.001
Adjusted average daily census	182 (89-267)	190 (108-312)	**210** (96-385)	197 (80-425)	69 (39-271)	<.001	240 (180-312)	**260** (163-398)	228 (117-419)	109 (60-255)	54 (39-126)	<.001

[1]Q1-Q3 represents the range of the lower quarter (Q1) to the upper quarter (Q3).
[2]Chi Square p-value reported to test for equal proportions of HCAHPS ratings. KrusKal-Wallis p-value reported to test for equal medians across HCAHPS rating categories.
[3]616 hospitals with missing data in HCAHPS sample for total facility FTE.

Patient Volume Indicators (Q1-Q3)[1]	Leapfrog Sample (N=1,824)											
	Spring Grade						Fall Grade					
	F	D	C	B	A	p-value	F	D	C	B	A	p-value
**Total Sample[2]**	**N=7**	**N=109**	**N=621**	**N=477**	**N=610**	<.001	**N=6**	**N=125**	**N=597**	**N=458**	**N=638**	<.001
Total facility admissions	7,018 (2,650-13,648)	9,807 (4,377-15,587)	8,920 (4,658-15,403)	9,350 (4,560-15,941)	9,264 (5,167-17,926)	0.483	3,563 (2,650-9,812)	**11,134** (5,924-17,196)	8,561 (4,368-15,159)	9,972 (5,078-17,034)	9,198 (5,084-16,492)	0.014
Adjusted admissions	12,217 (8,258-25,365)	20,457 (11,252-29,999)	19,686 (12,557-31,568)	20,779 (13,053-33,162)	20,957 (12,725-34,817)	0.181	9,918 (7,322-18,966)	**23,410** (12,718-35,085)	18,463 (12,149-30,350)	21,142 (13,213-34,054)	20,696 (12,725-33,765)	0.007
Total facility inpatient days	39,460 (35,814-69,693)	48,356 (21,833-89,784)	42,267 (21,636-76,514)	44,468 (20,226-80,565)	43,027 (21,836-82,405)	0.935	20,110 (11,628-40,423)	**57,877** (30,401-105,467)	40,285 (19,780-73,847)	47,929 (23,114-84,806)	40,900 (20,181-80,565)	0.002
Adjusted patient days	94,621 (65,904-129,525)	98,115 (59,582-181,737)	92,722 (56,435-155,484)	95,681 (53,309-175,387)	94,752 (55,099-166,800)	0.998	57,252 (31,912-84,071)	**119,644** (68,086-205,310)	90,344 (53,926-153,664)	100,995 (55,475-175,064)	91,260 (55,103-161,928)	0.002
Total facility Medicare discharges	3,705 (1349-5,974)	4,286 (2,056-6,935)	4,276 (2,392-7,586)	4,573 (2,347-7,646)	4,751 (2,627-7,922)	0.220	1,728 (1,349-4,038)	4,686 (2,954-8,160)	4,113 (2,296-7,138)	**4,709** (2,551-8,091)	4,661 (2,470-7,780)	0.019
Total facility Medicaid discharges	2,417 (520-3,437)	1,962 (813-4,307)	1,723 (802-3,541)	1,665 (764-3,206)	1,537 (784-3,303)	0.309	638 (409-1,932)	**2,305** (1025-4,858)	1,652 (767-3,321)	1,707 (837-3,386)	1,515 (745-3,162)	0.001
Total births	582 (0-2,362)	741 (322-1,507)	875 (398-1,731)	963 (384-1,928)	871 (360-2,111)	0.355	408 (0-664)	772 (266-1,973)	863 (386-1,695)	**1,055** (443-2,068)	847 (333-1,963)	0.032
Total surgical operations	4,115 (3,436-7,305)	6,194 (3,561-10,494)	6,672 (4,090-10,971)	6,923 (4,063-12,138)	6,788 (4,158-11,859)	0.222	2,958 (2,280-4,872)	7,064 (4,143-14,090)	6,235 (3,901-10,651)	**7,263** (4,448-12,246)	6,756 (4,136-11,553)	0.005
Emergency department visits	24,102 (21,612-46,387)	41,634 (26,158-69,237)	38,344 (25,305-59,391)	40,296 (25,918-62,206)	42,994 (27,620-65,297)	0.094	28,332 (21,612-40,085)	**43,698** (28,162-76,255)	37,004 (24,391-57,182)	43,013 (26,993-67,590)	41,251 (26,978-61,948)	0.011
Total facility personnel FTE[3]	1,197 (577-2,418)	1,285 (651-2,249)	1,116 (629-2,098)	1,186 (659-2,138)	1,152 (655-2,336)	0.988	483 (333-618)	**1,465** (740-2,576)	1,065 (627-2,043)	1,250 (725-2,398)	1,132 (601-2,072)	0.005
Adjusted average daily census	259 (181-355)	269 (163-498)	256 (156-428)	262 (146-481)	260 (151-457)	0.998	157 (87-230)	**328** (187-562)	248 (149-426)	277 (152-480)	250 (151-444)	0.002

[1]Q1-Q3 represents the range of the lower quarter (Q1) to the upper quarter (Q3).
[2]Chi Square p-value reported to test for equal proportions of Leapfrog grades. KrusKal-Wallis p-value reported to test for equal medians across Leapfrog grade categories.
[3]480 hospitals with missing data in Leapfrog sample for Total Facility FTE.

© 2022 See H et al. *JAMA Network Open.*
